# Dynamic Distribution of Gut Microbiota in Goats at Different Ages and Health States

**DOI:** 10.3389/fmicb.2018.02509

**Published:** 2018-10-24

**Authors:** Yujian Wang, Hao Zhang, Lin Zhu, Yulin Xu, Na Liu, Xiaomei Sun, Liping Hu, He Huang, Kai Wei, Ruiliang Zhu

**Affiliations:** ^1^Shandong Provincial Key Laboratory of Animal Biotechnology and Disease Control and Prevention, Shandong Agricultural University, Tai’an, China; ^2^Shandong Provincial Engineering Technology Research Center of Animal Disease Control and Prevention, Shandong Agricultural University, Tai’an, China; ^3^Animal Disease Prevention and Control Center of Shandong Province, Animal Husbandry and Veterinary Bureau of Shandong Province, Jinan, China; ^4^Shandong New Hope Liuhe Co., Ltd., New Hope Group, Qingdao, China

**Keywords:** Boer goats, gut microbiota, high-throughput sequencing, diarrheic kids, health states

## Abstract

The importance of the gut microbiota (GM) of animals is widely acknowledged because of its pivotal roles in metabolism, immunity, and health maintenance. The level of health can be reflected by the dynamic distribution of GM. In this study, high-throughput sequencing of the bacterial 16S rRNA gene was used to compare the microbial populations from feces in healthy and diarrheic kids, which reflected the dynamic shift of microbiota in kids and investigated differences from adult healthy goats. Healthy kids and goats not only displayed higher species richness but also exhibited higher bacterial diversity than diarrheic kids based on the results of the operational taxonomic unit analysis, alpha diversity, and beta diversity. Firmicutes and Bacteroidetes were the most dominant phyla in all samples. At the genus level, the differences in diversity and abundance between diarrheic kids and the other two groups were gradually observed. In the diarrheic kid intestine, Bacteroides remained the dominant species, and the proportion of Clostridium_sensu_stricto_1 and Paeniclostridium increased, whereas Rikenellaceae_RC9_gut_group, Ruminococcaceae_UCG-005, and Christensenellaceae_R-7_group were significantly reduced. The results showed the differences of GM in diarrheic kids and healthy kids were significant while in kids and goats were not obvious. Differences in the composition of intestinal microbiota may not be the cause of diarrhea, and some changes of bacterial richness may guide our interpretation of diarrhea. This study is the first to investigate the distribution of GM in Boer goats with different ages and health states. Furthermore, this study will provide a theoretical basis for the establishment of a prevention and treatment system for goat diarrhea.

## Introduction

As the largest and most complex mammalian micro-ecosystem, the gut microbiota (GM) not only regulates body health but also plays an important bridging role between diet and host (Turnbaugh et al., 2006; Wu and Wu, 2012). Studies indicate that the GM is a reflection of evolutionary selection pressures acting at the levels of the host and microbial cell. The GM is intimately involvedin numerous aspects of normal host physiology from nutritional status to behavior and stress response. Additionally, the GM serves as a central or contributing cause of many diseases, affecting both near and far organ systems (Ley et al., 2006; Mai and Draganov, 2009; Smith et al., 2011). This is especially true for ruminants, which have demonstrated their unique digestive properties and microbial groups that help to adapt to high fiber content foods, but also make them susceptible to a variety of diseases and conditions (Russell and Rychlik, 2001). Accordingly, GM in ruminants play a more prominent role in various physiological states compared to most other mammals (Chaucheyras-Durand and Durand, 2010; Rosenberg et al., 2010).

In animal husbandry, diarrhea in juvenile ruminants is a common and frequent disease associated with gastrointestinal dysfunction, which will affect their normal growth and even lead to their death. Several studies have demonstrated that some GM of ruminants have been alternating between dominant and weak populations accompanied by the occurrence of diarrhea (Steele et al., 2015; Malmuthuge and Guan, 2016; Tarabees et al., 2016). Thus, some inevitable associations may be present between changes in GM and the occurrence of diarrhea in ruminants. However, the specific connections, changing characteristics, and laws are not known. In addition, some potential links may also exist between age factors and intestinal microbiota. However, whether the participation of age-related factors will affect the intestinal microecology of ruminants has been rarely reported at present.

Recently, new technologies based on high-throughput sequencing have been developed and successfully applied to the analysis of the complex bacterial ecosystem of the gut (Glenn, 2011; Kim and Isaacson, 2015). By deeply analyzing and comparing the information obtained, the mechanisms contributing to ill health can be further understood, and strategies can be developed to ensure that the collateral damage inflicted is minimal (Cotter et al., 2012; Zhou J. et al., 2015). Boer goats are widely raised in China because of their high quality, which is connected not only with their own genes but also probably with intestinal microorganisms. However, to date, the relationship between the composition of the GM in goats and diarrhea is not very clear. In this study, we used the high-throughput sequencing approach to investigate the microbiota composition of stool samples from diarrheic kids, healthy kids, and adult goats. In the comparison of diarrheic kids and matched health controls, a significant difference was observed in the microbiota composition ratio and the proportion of some bacterial populations. Notably, the comparison of microflora among different age groups was unexpected, and the diversity of GM between adult goats and kids was almost similar.

## Materials and Methods

### Ethics Statement

The animal experiments were approved by Animal Protection and Utilization Committee of Shandong Agricultural University (Permit number: 20010510) and executed in accordance with Guide to Animal Experiments of Ministry of Science and Technology (Beijing, China). This study did not involve any endangered or protected species.

### Animals and Sample Collection

A total of 15 Boer goats were obtained from a commercial feedlot (Shandong Province, China), including five diarrheic kids (2–3 months old), five healthy kids (2–3 months old), and five healthy adult goats (7–8 months old), were used in this experiment. The ratio of male to female in each group is 3:2. The Boer goats we screened were self-propagated and raised by the commercial goat farm and had similar genetic backgrounds. All selected animals used the same immune procedure and no other illnesses occurred prior to the sample collection. The animals were fed with standard goat diet under the same husbandry. Table 1 provides the ingredients of the diets. One day prior to sample collection, all animals were placed in a dedicated area of the commercial feedlot and maintained a normal diet. Two or three separate fecal samples were collected from each animal using sterile tool the following morning. Freshly rectal feces were selected and sub-sampled (approximately 100 g) from the central portion to minimize contamination by bedding and flooring and then stored in sterile plastic containers at -20°C. All samples (healthy kids, G1, G2, G3, G4, and G5; diarrheic kids, GF1, GF2, GF3, GF4, and GF5; and healthy adult goats, C1, C2, C3, C4, and C5) were transported to the laboratory within 2 h in ice and later stored at -80°C.

**Table 1:** Composition of ingredients for goat feed.

Diet type^1^	Adult goat	Kid
Ingredient (g)		
Corn	90	325
Bran	30	50
Alfalfa meal	300	150
Wild hay powder	500	
Corn straw powder	200	350
Iodized salt	10	10
Selenium trace elements additives	6	
Soybean meal	30	45
Sunflower meal		50
Trace elements and vitamin premix^2^	30	50


*^*1*^Manufacturer: Beijing Sanyuan Hefeng Farming Co., Ltd., Beijing, China. ^*2*^Detailed supplementation not disclosed by the manufacturer.*

### DNA Extraction

Microbial genomic DNA was extracted from 500 mg of each fecal sample using a TIANamp Genomic DNA kit (TIANGEN Biotech Co., Ltd., Beijing, China) following the manufacturer’s instructions. The quality of the DNA was examined by 0.8% (w/v) agarose gel electrophoresis and the concentration measured with a UV-Vis spectrophotometer (NanoDrop 2000, United States).

### PCR Amplification of 16S rDNA

Next generation sequencing library preparations and Illumina MiSeq sequencing were conducted at GENEWIZ, Inc. (Suzhou, China). 30–50 ng DNA was used to generate amplicons using a MetaVx^TM^ Library Preparation kit (GENEWIZ, Inc., South Plainfield, NJ, United States). V3, V4, and V5 hypervariable regions of prokaryotic 16S rDNA were selected for generating amplicons and following taxonomy analysis. GENEWIZ designed a panel of proprietary primers aimed at relatively conserved regions bordering the V3, V4, and V5 hypervariable regions of bacteria 16S rDNA. The V3 and V4 regions were amplified using forward primers containing the sequence “CCTACGGRRBGCASCAGKVRVGAAT” and reverse primers containing the sequence “GGACTACNVGGGTWTCTAATCC.” The V4 and V5 regions were amplified using forward primers containing the sequence “GTGYCAGCMGCCGCGGTAA” and reverse primers containing the sequence “CTTGTGCGGKCCCCCGYCAATTC.” The PCR was run in a total reaction volume of 25 μL. Each reaction mixture contained 2.5 μL 10× PCR buffer, 2 μL dNTP (10 mM each), and 0.5 μL Taq DNA polymerase (5 U/μL; TransGen Biotech, TransStart, China), forward and reverse primers (1 μL each, 50 μM), 2 μL DNA template and sterile water. PCR was performed under the following conditions: 94°C for 3 min followed by 24 cycles of 94°C for 5 s, 57°C for 90 s and 72°C for 10 s, and a final elongation step at 72°C for 5 min. At the same time, indexed adapters were added to the ends of the 16S rDNA amplicons to generate indexed libraries ready for downstream NGS sequencing on Illumina Miseq. DNA libraries were multiplexed and loaded on an Illumina MiSeq instrument according to manufacturer’s instructions (Illumina, San Diego, CA, United States). Sequencing was performed using a 2 × 250 or 2 × 300 paired-end configuration. The sequences of V3, V4, and V5 were processed, spliced, and analyzed by GENEWIZ (Beijing, China).

### Bioinformatics and Statistical Analysis

The QIIME (Qiime1.9.1) data analysis package was used for 16S rRNA data quality control and analysis. Short reads (<200 bp) and low quality phred (average quality score <20) were discarded. The SILVA bacterial database was used to align the resulting sequences. The pre.cluster and chimera.uchime commands of Mothur (Schloss et al., 2009) were used to detect and remove chimera sequences. The effective sequences were used in the final analysis. Sequences were grouped into operational taxonomic units (OTUs) using the clustering program VSEARCH (1.9.6.) against the Silva 119 database pre-clustered at 97% sequence identity. The Ribosomal Database Program (RDP) classifier was used to assign taxonomic category to all OTUs at confidence threshold of 0.8. Sequences were rarefied prior to calculation of alpha and beta diversity statistics. Alpha diversity indexes were calculated in QIIME from rarefied samples using for diversity the Shannon index, for richness the Chao1 index. Beta diversity was calculated using weighted and unweighted UniFrac and principal coordinate analysis (PCoA) performed. Unweighted Pair Group Method with Arithmetic mean (UPGMA) tree from beta diversity distance matrix was builded. The criterion of significance was conducted at *P* < 0.05. Data were expressed as mean ± standard deviation (SD) and were performed using SPSS 17.0. Raw sequence data of this study have been deposited to the NCBI Sequence Read Archive with accession No. PRJNA 436893.

## Results

### DNA Sequence Data and Microbial Diversity Index Analysis

**FIGURE 1:**
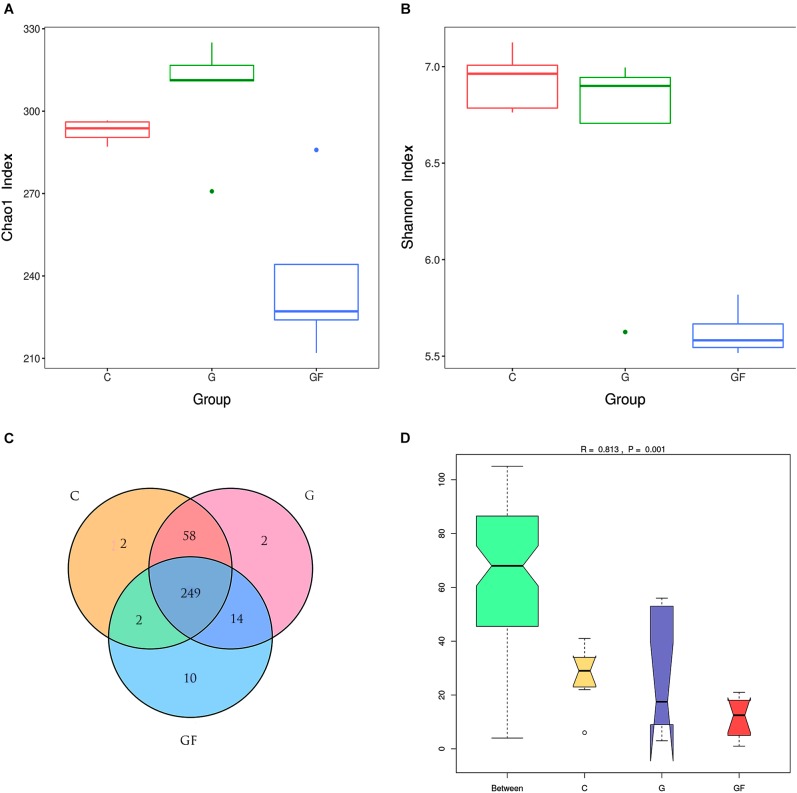
DNA sequence data and microbial diversity index analysis. **(A)** Chao1 index. **(B)** Shannon index. **(C)** Venn diagram. The numbers in the figure represent the unique or common OTUs of each group. **(D)** ANOSIM analysis. “Between” represents the difference between the three groups, the closer the *R*-value is to 1, the greater the difference between the groups. “C,” “G,” and “GF” represent the different three groups.

A total of 2,291,270 pairs of 300 bp readings were obtained, and 668, 126, 841, 320 and 782,824 raw readings in the C, G, and GF groups, respectively. After optimizing the original data, 886,467 high-quality valid sequences were obtained. On the basis of 97% species similarity, 311, 323, and 275 OTUs were separately obtained from samples at groups C, G, and GF (Table 2), respectively. A total of 338 OTUs were identified from all samples, of which 249 exist in all groups defined as core OTUs (Figure 1). The core OTUs comprised approximately 73.6% of the total OTUs. In addition, 2 OTUs were uniquely identified in both C and G groups, and 10 unique OTUs were found in group GF.

**Table 2:** Sequence data of the samples.

Group	Raw reads	High quality valid sequences	OTUs	Average valid sequences of sample
C	668126	265433	311	53086
G	841320	309817	323	61963
GF	782824	311217	275	62243


The multiple α-diversity indices were used to analyze the Community richness and diversity. In accordance with the Chao1 estimator, 293, 300, and 234average OTUs were shown in samples at groups C, G, and GF, respectively, whereas the ACE estimator showed 292, 306, and 238, which suggested the abundance of GM. The Shannon–Wiener index could directly reflect the heterogeneity of a community based on the number of species present and their relative abundance (López-González et al., 2015). The Shannon–Wiener indices of groups C, G, and GF were 6.92, 6.63, and 5.62, respectively. Intergroup analysis of Chao1 and Shannon index intuitively showed that the abundance and diversity of bacterial community in the GF group were lower than those in groups C and G (*P <* 0.05), whereas the difference between the C and G groups was insignificant (Figure 1). Consistently, a lower Simpson diversity index was found in the GF group than in the C and G groups. Furthermore, ANOSIM showed that differences between groups were greater than those within groups (*R* = 0.813, *P* < 0.001; Figure 1). In addition, Good’s coverage estimates were 99.9, 99.8, and 99.9% for C, G, and GF groups, respectively, all showing excellent coverage.

**FIGURE 2:**
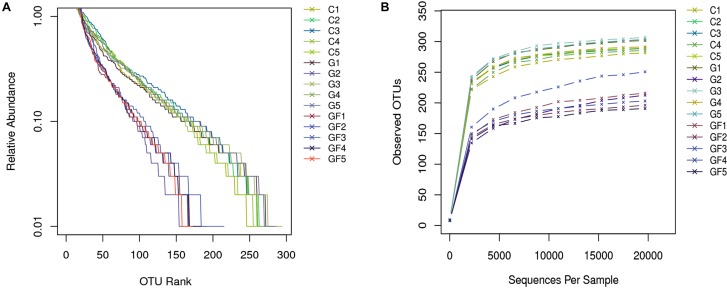
Sample feasibility analysis. Each curve represents a sample. **(A)** Rank-Abundance curve. The abscissa indicates the OTU (species) abundance order, and the ordinate corresponds to the relative abundance ratio of OTU (species). **(B)** Rarefaction curves depicting the effect of sequences on the number of OTUs identified.

**FIGURE 3:**
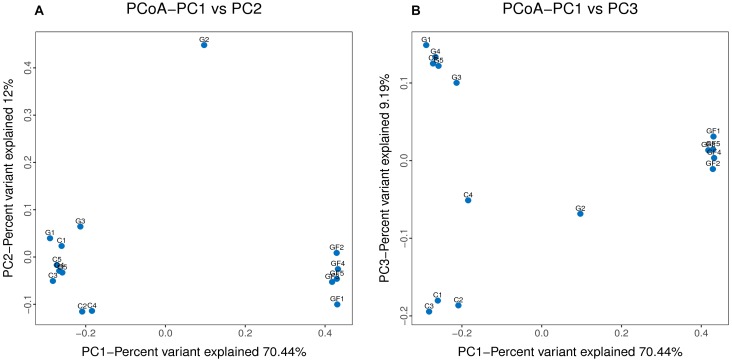
Differences in bacterial community structures. **(A,B)** Principal Coordinate Analysis (PCoA) of bacterial community structures of the GM in the three sample groups. Each blue point represents each sample. The distance between the two points represents the difference of GM.

The rank abundance curve further demonstrates species abundance and evenness. In all samples, the OTU Ranks were within 300, indicating that species compositions of the each sample were less abundant. All curves are relatively flat, indicating that species compositions of all samples were relatively uniform (Figure 2). Moreover, as shown in Figure 2B, the curve tends to be flat when the number of effective sequences reaches 20,000. In this study, the number of valid sequences of each sample was more than 50,000, which indicated that the amounts of sequencing data were sufficient. The PCoA of the UniFrac distance matrix clearly showed the differences among all sample individuals or groups. The microbiota in the GF group clustered along the principal coordinates 1 and 2 and separated from the other two groups of microbiota (Figure 3). In addition, with the exception of G2 and C5, the samples in each group clustered separately, indicating that the differences in the communities within the groups were small, whereas the G2 and C5 were specific (Figure 3).

### Bacterial Community Composition at Different Taxonomical Levels

**FIGURE 4:**
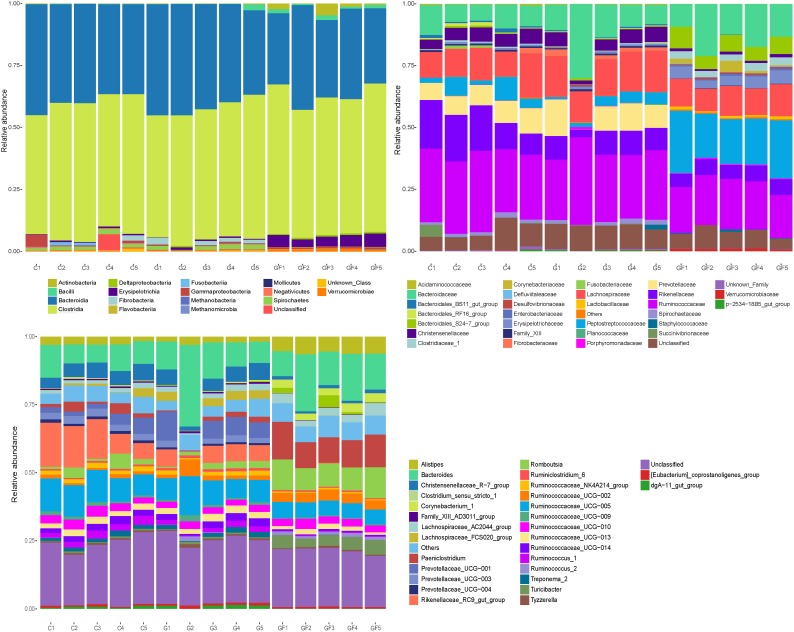
Microbial composition of different samples. Each bar represents the average relative abundance of each bacterial taxon within a group. **(A)** Taxa assignments at Phylum level. **(B)** Taxa assignments at Family level. **(C)** Taxa assignments at Genus level.

We analyzed the gut bacterial community composition and structure in different taxonomical levels. In accordance with the phylum assignment result, Firmicutes was the predominant phylum in the 15 samples, whereas Bacteroidetes was secondary. The high abundance of phylum Actinobacteria was found in GF1, GF3, and GF4 samples, and Fibrobacteres was found in C5, G1, G3, G4, and G5. Interestingly, Fusobacteria was found only in the C2 and C3 samples (Figure 4). Besides the phylum, bacterial abundance was also analyzed specifically at other taxonomic units, family, and genus (Figure 4).

On the family level, a total of 31 families were identified from all samples. As shown in Figure 4, no significant differences were observed between the C and G groups except the G2 sample. Ruminococcaceae, Bacteroidaceae, Lachnospiraceae, Rikenellaceae, and Prevotellaceae were the most abundant in both C and G groups, whereas Prevotellaceae was almost absent in the G2 samples. Moreover, the most abundant families in the GF group were Peptostreptococcaceae, Ruminococcaceae, Bacteroidaceae, Lachnospiraceae, Bacteroidales_S24-7_group, Rikenellaceae, and Enterobacteriaceae. In comparison with the other two groups, Peptostreptococcaceae, Bacteroidales_S24-7_group, Enterobacteriaceae, and Clostridiaceae_1 increased significantly in the GF group, whereas Prevotellaceae and Christensenellaceae decreased significantly (Figure 4).

On the genus level, a total of 31 genera were identified from all samples. Similar to the family level, no significant differences were observed between the C and G groups except the G2 sample. The predominant genera in groups C and G (except G2) included *Rikenellaceae_RC9_gut_group*, *Ruminococcaceae_UCG-005*, and *Bacteroides*. However, in the G2 sample, *Bacteroides*, *Ruminococcaceae_UCG-005*, and *Ruminococcaceae_UCG-002* were relatively abundant. Moreover, in the GF group, *Bacteroides* remained the predominant population. However, it should be noted that both the relative and absolute abundance of *Paeni Clostridium*, *Clostridium_sensu_stricto_1*, and *Romboutsia* increased significantly while *Rikenellaceae_RC9_gut_group*, *Prevotellaceae_UCG-001*, and *Ruminococcaceae_UCG-005* were opposite (Figure 4).

The important result was that at the level of families and genera, the differences in diversity and abundance between the GF group and the other two groups were gradually discovered, which was consistent with the results of the previous analysis. Moreover, the differences in population diversity between C and G groups were minimal, but the abundance of some populations changed with age.

### Correlation of GM With Health Level and Age Difference

**FIGURE 5:**
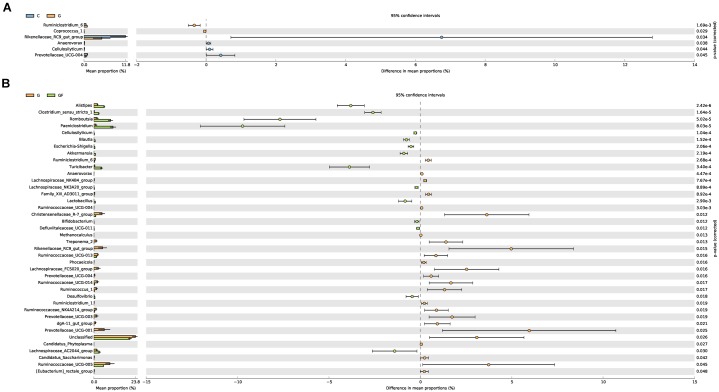
Differences in bacterial abundance between the groups. The left side of the graph shows the abundance ratios of different strains in two samples. The middle graph shows the difference in bacterial abundance within the 95% confidence interval. The rightmost values are the *P*-values of the significance test. **(A)** Differences in species abundance between groups G and C. **(B)** Differences in species abundance between groups G and GF.

Significant differences were noted in the diversity and abundance of intestinal microbiota with changed age and health status. To investigate the age and health difference in GM, we performed a difference analysis of two aspects by using STAMP (Figure 5). As shown in Figure 5A, there were no obvious differences in other GM between different age groups except for *Rikenellaceae RC9 gut_group* (*P* < 0.05). The results shown in Figure 5B describe the differences of the GM between healthy and diarrheic kids, explaining the health-related association of GM. *Alistipes* and *Ruminococcaceae_UCG-005* were previously determined to have a high abundance in both G and GF groups, indicating that these are necessary for kids (Figure 5). However, *Prevotellaceae_UCG-001*, *Paeniclostridium*, *Christensenellaceae_R-7_group*, *Romboutsia*, *Rikenellaceae_RC9_gut_group*, and *Ruminococcaceae_UCG-005* showed significant differences (*P* < 0.05). Compared with group G, *Rikenellaceae RC9_gut_group, Prevotellaceae UCG-001*, and *Ruminococcaceae UCG-005* in GF group were significantly reduced, and *Pani Clostridium* and *Romboutsia* were significantly increased. In addition, *Treponema 2, Lachnospiraceae FCS020_group, Ruminococcus 1, Ruminococcaceae UCG-014, Prevotellaceae_UCG-003*, and *dgA-11_gut_group* in the G group also showed an increasing trend, their abundance is low but they cannot be ignored.

## Discussion

To date, researches into mammalian intestinal microbiota have covered many aspects, including metabolism, physiology, and immunology (Clemente et al., 2012; Zeng et al., 2015), but few reports have been published on the differences of the GM in different health states and ages of goats. In this study, we analyzed the bacterial diversity and abundance of rectal contents of Boer goats in different health states and ages. Results showed that the bacterial abundance and diversity in diarrheic kids were lower than those in healthy goats, whereas the differences of the intestinal microbiota between adult goats and kids were not significant.

Age has always been speculated to be an important factor affecting human and animals GM. Several studies indicate that the GM of mammals were normally influenced by genotype or gender during development and reached stability at maturity (Poroyko et al., 2011; David et al., 2014; Zhao et al., 2015). Jami et al. (2013) observed that the rumen microbial diversity of cattle increased and a convergence toward a mature bacterial arrangement with age. Hu et al. (2017) indicated that the musk deer gut environment developed into a more restricted niche within the host as the animal aged. However, in our study, we found that the differences of microbial diversity between adult goats and kids were not significant (only *Rikenellaceae RC9 gut_group* is different). The microbial diversity of Boer goats did not change significantly with age, which is inconsistent with previous observations in ruminants (Jami et al., 2013; Hu et al., 2017). We speculated that there may be differences in the composition and development of GM among different species, while the GM of goats may reach a steady state at an earlier age. Interestingly, although the differences in microbial diversity between different age groups were not significant, the proportion of some intestinal microbiota changed. Compared with kids, the proportion of *Prevotellaceae_UCG-001* and *Lachnospiraceae_FCS020_group* in the intestinal microbiota of adult goats increased, whereas the ratio of *Rikenellaceae_RC9_gut_group* and *Ruminococcaceae_UCG-005* decreased. This may be the result of the host’s evolution toward a better structure during development (Jami et al., 2013). It is worth noting that the age difference between kids and adult goats in this study is about 4–5 months. Perhaps the bigger age gap will reflect more differences of GM, which require further verification.

Health state is inevitably related to ages. The incidence of lamb diarrhea is extremely high, and this condition gradually improves with age. Intestinal microfloras have been shown to be closely linked to diarrhea (Türkyılmaz et al., 2014). However, our study showed that intestinal microfloras were not significantly different in different age groups. Therefore, we suspect that the main reason why kids are more prone to diarrhea than adult goats may not be the difference in composition of GM. Some other factors, such as intestinal mucosal developmental immaturity, low pH in the gastric juice, and the external environment may make the kids more susceptible to pathogen invasion (Causapé et al., 2002; Andrés et al., 2007). The invasion of foreign pathogens causes the lamb to develop diarrhea, which in turn causes dynamic changes in the intestinal microbiota.

The factors that cause diarrhea are varied, and our specimen collection has avoided the outbreak period of a particular pathogen, ensuring that the samples we collected came from general diarrhea. We further explored the GM of this common diarrhea of goats. The differences of specific bacteria intuitively reveal the intrinsic relationship between GM and kid diarrhea. Our study found that Firmicutes and Bacteroidetes were the most dominant phyla, regardless of age and health status, representing approximately 92% of the total sequences. These results were similar to what others have observed in sheep and other ruminants (Whitehead and Cotta, 2001; Sundset et al., 2007; Kong et al., 2010; Samsudin et al., 2011). For ruminants, Firmicutes plays an important role in degrading fiber and cellulose (Thoetkiattikul et al., 2013), while the main function of Bacteroidetes is to degrade carbohydrates and proteins, and facilitate the development of gastrointestinal immune system (Spence et al., 2006; Meital et al., 2016). In addition, a high variation could be observed at diarrheic kids for other important phyla, especially for Actinobacteria and Verrucomicrobia. Interestingly, Actinobacteria synergy with one partner or host can easily be translated into pathogenic interactions with another (Miao and Davies, 2010).

At the genus level, Bacteroides remained the dominant species in diarrheic kids and had a tendency to increase. *Bacteroides*, as a normal GM in the gut of ruminants, can cause an endogenous infection when the immune system or intestinal microbiota is dysfunctional. Remarkably, the percentage of *Paeniclostridium*, *Clostridium_sensu_stricto_1*, *Turicibacter*, and *Romboutsia* in diarrheic kids was significantly increased compared with healthy populations. *Clostridium* has long been thought to be closely related to diarrhea and intestinal toxemia in ruminants, and its toxins affect the body through different pathways (Lewis and Naylor, 1998). The lethal toxin factor produced by *Paeniclostridium* can binds to the host and affects the normal glycosylation reaction (Ziegler et al., 2008). *Clostridium_sensu_stricto_1* has also been shown to play a key role in causing necrotizing enterocolitis in preterm infants (Zhou Y. et al., 2015). The pathogenicity of *Turicibacter* has not been clarified, however, in some studies it has been found that the abundance of *Turicibacter* increases during enteritis (Bretin et al., 2018). Moreover, it has been reported that the increase of bile salt hydrolase and urease enzymes in intestinal diseases provides an environment for the survival of *Romboutsia* (Gerritsen et al., 2017). Obviously, these colonies share common characteristics: (a) The abundance increased with diarrhea; (b) Adapt, maintain and even promote diarrhea. By contrast, the percentages of *Rikenellaceae_RC9_gut_group*, *Ruminococcaceae_UCG-005*, and *Christensenellaceae_R-7_group* were significantly reduced. *Rikenellaceae* are correlated with resistance to the development of colitis following CTLA-4 blockade and can limit inflammation by stimulating T-regulatory cell differentiation (Dubin et al., 2016). *Ruminococcaceae* and *Christensenellaceae* regarded as potential beneficial bacteria because they participated in the positive regulation of the intestinal environment and linked to immunomodulation and healthy homeostasis (Kong et al., 2016; Shang et al., 2016). This conveys a message that diarrhea leads to a decrease in beneficial bacteria, or that the reduction in beneficial bacteria exacerbates diarrhea. As mentioned in the previous analysis, there was no significant difference in intestinal microbiota in different age groups, whereas significant changes occurred in the diarrhea group, which made us more convinced that the occurrence of diarrhea caused obvious dynamic changes in intestinal microbiota. The pathogenic bacteria are increasing and the probiotics are decreasing. If some measures are taken, making a large increase in beneficial bacteria or a large reduction in pathogenic bacteria, may have a positive therapeutic effect on diarrhea.

In this work, our samples were mainly from the rectal contents of goats. Due to the fermentation in the hindgut segments of mammals (Caporaso et al., 2010), researchers should generally refrain from extrapolating fecal inventories as indicators of microbial diversity of specific gut regions. However, fecal inventories are still useful to researchers. Fecal inventories are informative when comparing treatment groups or species within a study, and provide the opportunity to conduct repeated sampling of an individual or collect non-invasive samples (Kohl et al., 2014). The primary objective of this study is to guide the diagnosis of goat disease by analyzing the intestinal microbiota of goats of different ages and health status. In addition, it also provides a theoretical basis for the development of intestinal microecological preparations.

It is well established that diarrhea involve a multifactorial disease, and the microbiota is just one of several factors in the pathogenesis of the disease. However, we believe that microbes can better reflect the health status of animals. Future studies will evaluate the parameters of GM after treatment as a comparison for microbiota dysbiosis associated with goat diarrhea, and the usefulness of tracking microbiota over time and in response to treatment. When more validated treatment options become available, the study on analysis of intestinal microbes in different health states may help the clinician to judge whether the microbiota returns to a normal state. Of course, this requires further exploration and research.

## Conclusion

Taken together, our study found that the GM in diarrheic goats undergoes drastic changes, and there is a significant difference in the composition of GM between diarrhea kids and healthy kids. Conversely, the difference in composition of GM between healthy kids and adult goats is not obvious. Compared with adult goats, lambs are more susceptible to diarrhea, which may have little to do with the composition of GM, and are more likely to be caused by other factors. However, for kids, once diarrhea occurs, for whatever reason, it may significantly affect the state of the GM which will exacerbates intestinal dysfunction. In addition, we have also found that some bacteria, such as *Clostridium_sensu_stricto_1*, *Paeniclostridium*, *Rikenellaceae_RC9_gut_group*, and *Ruminococcaceae_UCG-005*, can serve as the signal of conventional diarrhea, which their abundance obviously increased or decreased after diarrhea. These findings can also be regarded as a normal instruction for evaluating intestinal health.

## Author Contributions

RZ, KW, LH, YW, and HZ designed the research. YW, HZ, LZ, YX, XS, and NL performed the research. YW, HZ, LH, KW, and RZ analyzed the data and wrote the paper.

## Conflict of Interest Statement

The authors declare that the research was conducted in the absence of any commercial or financial relationships that could be construed as a potential conflict of interest.
